# Molecular typing of *Leptospira interrogans* serovar Hardjo isolates from leptospirosis outbreaks in Brazilian livestock

**DOI:** 10.1186/s12917-017-1081-9

**Published:** 2017-06-15

**Authors:** Maria Raquel V. Cosate, Tetsu Sakamoto, Tiago Antônio de Oliveira Mendes, Élvio C. Moreira, Carlos G. Regis da Silva, Bruno S. A. F. Brasil, Camila S. F. Oliveira, Vasco Ariston de Azevedo, José Miguel Ortega, Rômulo C. Leite, João Paulo Haddad

**Affiliations:** 1 Instituto de Ciências Biológicas, Departamento de Bioquímica e Imunologia, Universidade Federal de Minas Gerais, Belo Horizonte, MG Brazil; 2 Laboratório de Biologia Parasitária, Centro de Pesquisas Gonçalo Moniz- Fiocruz, Salvador, BA Brazil; 3 Departamento de Medicina Veterinária Preventiva, Escola de Veterinária, Universidade Federal de Minas Gerais, Belo Horizonte, MG Brazil; 4 Embrapa Agroenergia, Brasília, Brazil; 5 Universidade Federal de Minas Gerais, Departamento de Genética - Instituto de Ciências Biol’ogicas de Minas Gerais, Belo Horizonte, Brazil

## Abstract

**Background:**

Leptospirosis is caused by pathogenic spirochetes of the genus *Leptospira* spp. This zoonotic disease is distributed globally and affects domestic animals, including cattle. *Leptospira interrogans* serogroup Sejroe serovar Hardjo and *Leptospira borgpetersenii* serogroup Sejroe serovar Hardjo remain important species associated with this reproductive disease in livestock production. Previous studies on Brazilian livestock have reported that *L. interrogans* serovar Hardjo is the most prevalent leptospiral agent in this country and is related to clinical signs of leptospirosis, which lead to economic losses in production. Here, we described the isolation of three clinical strains (Norma, Lagoa and Bolivia) obtained from leptospirosis outbreaks that occurred in Minas Gerais state in 1994 and 2008.

**Results:**

Serological and molecular typing using housekeeping (*secY* and 16SrRNA) and *rfb* locus (ORF22 and ORF36) genes were applied for the identification and comparative analysis of *Leptospira* spp. Our results identified the three isolates as *L. interrogans* serogroup Sejroe serovar Hardjo and confirmed the occurrence of this bacterial strain in Brazilian livestock. Genetic analysis using ORF22 and ORF36 grouped the *Leptospira* into serogroup Sejroe and subtype Hardjoprajitno. Genetic approaches were also applied to compare distinct serovars of *L. interrogans* strains by verifying the copy numbers of the IS1500 and IS1533 insertion sequences (ISs). The IS1500 copy number varied among the analyzed *L. interrogans* strains.

**Conclusion:**

This study provides evidence that *L. interrogans* serogroup Sejroe serovar Hardjo subtype Hardjoprajitno causes bovine leptospirosis in Brazilian production. The molecular results suggested that *rfb* locus (ORF22 and ORF36) could improve epidemiological studies by allowing the identification of *Leptospira* spp. at the serogroup level. Additionally, the IS1500 and IS1533 IS copy number analysis suggested distinct genomic features among closely related leptospiral strains.

**Electronic supplementary material:**

The online version of this article (doi:10.1186/s12917-017-1081-9) contains supplementary material, which is available to authorized users.

## Background

Leptospirosis is one of the most significant bacterial zoonotic diseases. It is caused by pathogenic species of the genus *Leptospira* and results in high mortality and morbidity [1, 2]. Rodents are the main reservoirs of spirochetes, and different species can function as maintenance hosts. Humans are usually infected through contaminated water or direct contact with the infected urine or abortion fluids of rodents and livestock animals [3, 4]. The most prevalent subtypes in cattle herds are *Leptospira borgpetersenii* serovar Hardjo and *Leptospira interrogans* serovar Hardjo. Differences among these subtypes are related to the geographic areas of isolation, genomic patterns and pathogenic features [5]. Hardjoprajitno isolates have been described in Australia, the United States of America (USA), the United Kingdom, Mexico, and Canada [6]. In South America, this *Leptospira* subtype was first reported in Argentina [7], and serological analyses showed a high prevalence of serogroup Sejroe in livestock in Brazil [8].

The identification and characterization of new bacterial strains have enhanced epidemiological studies of disease and improved general knowledge of the isolates [4]. However, the *Leptospira* genus presents a different taxonomic classification among the bacterial phyla, with both its serological and genetic features useful for phylogenetic identification [9]. *Leptospira* spp. taxonomic classification involves genomic features and serological analyses and comprises approximately 300 serovars grouped into 24 serogroups and several species. In the clinical setting, microscopic agglutination testing (MAT) is the serologic method used for leptospirosis diagnosis. However, because this technique is difficult and laborious, new molecular approaches are relevant candidates for improvement of the classification of *Leptospira* species isolates in routine samples.

Bovine leptospirosis infections cause clinical conditions associated with reproductive failure, including abortion, mastitis and a reduction in milk production [10]. The pathogenic and clinical evolution of these infections could be associated with the *Leptospira* subtype [11]. Serological characteristics are based on differences in the structure of the exposed lipopolysaccharide (LPS), which is synthesized by proteins at the *rfb* locus [12–14]. This locus was first identified in the *L. interrogans* serovar Copenhageni [12]. Subsequent studies reported that *L. borgpetersenii* serovar Hardjo contained 32 open read frames (ORFs) and serovar Copenhageni contained 31 ORFs inside the *rfb* locus [13, 14]. An additional ORF found in *L. interrogans* serovar Hardjo was named ORF36 [15]. Previous analyses suggested that ORF1-ORF14 and ORF21-ORF22 of the *rfb* locus in *L. interrogans* serovar Hardjo were acquired by lateral gene transfer from *L. borgpetersenii* serovar Hardjo and that ORF15-ORF20 and ORF23-ORF31 were acquired by lateral gene transfer from *L. interrogans* serovar Copenhageni [15].

Different methodologies have been applied in *Leptospira* spp. genetic studies to improve the characterization of leptospirosis, including quantitative real-time PCR techniques, which have been used for diagnostic and taxonomic purposes and the comparative analysis of *Leptospira* strains for epidemiological studies [16, 17]. The determination of insertion sequences and copy numbers has also been used for molecular bacterial typing [18, 19, 20]. For instance, the IS1500 and IS1533 mobile elements, which are classified in the IS3-like and IS110 families, respectively, were more frequent in the genomes of the major pathogenic species *Leptospira interrogans* and *Leptospira borgpetersenii*, respectively [21–23].

In the present study, we describe three clinical strains of *Leptospira* spp. from two leptospirosis outbreaks in livestock that occurred in the state of Minas Gerais, Brazil, in 1994 and 2009. To identify the isolates, we performed MAT and applied genotyping approaches using housekeeping (*secY* and 16S rRNA) and *rfb* locus (ORF22 and ORF36) genes. Furthermore, real-time quantitative PCR was performed to compare the relative copy numbers of IS1500 and IS1533 among the clinical and reference leptospiral strains. Our data suggested that *rfb* loci (ORF22 and ORF36) could be useful for serovar and serogroup identification and that mobile elements might indicate differences among *Leptospira* strains.

## Methods

### *Leptospira* spp. reference strains

We used 14 *Leptospira* strains belonging to *Leptospira interrogans* and *Leptospira borgpetersenii* for the genotyping analysis (Table 1). The reference strains were obtained from the Pan American Health Organization, Argentina and the Royal Tropical Institute, the Netherland, Holland, Amsterdam. The *Leptospira* spp. were cultured in liquid Ellinghausen and McCullough (EMJH) medium modified under aerobic conditions at 28 °C in the absence of light [9].

**Table 1:** List of *Leptospira* spp. reference strains

Species	Serogroup	Serovar	Strain
*L. interrogans*	Canicola	Canicola	Hond Utrecht IV
	Pomona	Pomona	Pomona
	Icterohaemorrhagiae	Lai	Lai
	Icterohaemorrhagiae	Copenhageni	M-20
	Bataviae	Bataviae	Van Tienen
	Sejroe	Hardjo	Hardjoprajitno (OMS)
	Hebdomadis	Hebdomadis	Hebdomadis
	Australis	Bratislava	Jez Bratislava
	Sejroe	Wolffi	Wolffi
*L. borgpetersenii*	Tarassovi	Tarassovi	Perepelicin
	Mini	Mini	Sari
	Sejroe	Sejroe	M-84
	Sejroe	Hardjo	Hardjobovis-Sponselee
	Mini	Swazizak	Swajizak

### Epidemiological characterization of leptospirosis outbreaks

Blood and urine were collected from 316 and 326 animals, respectively, from two dairy farms in the state of Minas Gerais, Brazil, where leptospirosis outbreaks in livestock occurred in May 1994 and May 2009. All of the selected animals presented clinical signs compatible with leptospirosis, such as abortions, reduction in milk production and stillbirths [10]. The collected blood samples were submitted to MAT [24]. We collected the urine of MAT-positive animals to proceed with *Leptospira* isolation.

### Microscopic agglutination test (MAT)

To confirm the leptospirosis diagnosis, MAT was used to detect antibodies to *Leptospira* according to the method of Faine et al. [9]. MAT was also used for serological bacterial typing of isolates according to the Leptospirosis Reference Centre (KIT, the Netherlands). Live cultures of 13 *Leptospira* spp. reference strains representing eight *L. interrogans* serogroups and four *L. borgpetersenii* serogroups (sensu *lato* qualification) were used (Additional file 1). The sera were screened at 1:100 dilutions and further diluted on a two-fold basis to determine the final titer. *Leptospira* spp. interpretation based on the MAT was performed by the identification of an agglutination index ≥50% per microscopic field at a given dilution, which was considered a positive reaction.

### *Leptospira* spp. isolation procedure

Ten-fold serial dilutions of urine samples directly obtained from MAT-positive farm animals were inoculated into tubes containing 5 ml of EMJH medium supplemented with 10% bovine serum albumin and 5% rabbit serum. The cultures were incubated at 28 °C and examined weekly for 10 weeks using dark-field microscope [9]. Five hamsters were inoculated intraperitoneally with 500 μl of positive culture. Their kidneys were obtained 21 days after inoculation and cultured in EMJH medium [9]. Clinical isolates were typed with the monoclonal antibodies (mABs) F16C28 and F106C1, which were specific for serogroup Sejroe and serovar Mini, respectively, in the agglutination reaction. The monoclonal antibodies were produced and screened at the Royal Tropical Institute (KIT), Amsterdam, the Netherlands.

### DNA isolation, conventional PCR and gene sequencing

A total of 10^6^ cells were collected by centrifugation at 13,000 g and incubated overnight at 50 °C in 200 μl of lysis buffer (50 mM Tris/HCl, pH 8.0, 50 mM EDTA, 100 mM NaCl, and 1% SDS) containing 100 μg/ml proteinase K. Genomic DNA was extracted by the phenol/chloroform method and precipitated with ethanol [25].

PCR was performed with a total volume of 25 μl, including 200 μM dNTPs, 1.5 mM MgCl_2,_ 10 mM KCl, 10 mM Tris-HCl, 10 pM of each primer, 20 ng of genomic DNA and 500 U of Taq polymerase. The primer sequences and the expected product sizes are shown in Table 2. The reaction mixtures were subjected to the following amplification protocol for all of the genes analyzed: 3 min at 95 °C, followed by 29 cycles consisting of 95 °C for 1 min, a target-specific annealing temperature for 1 min and 72 °C for 1 min, and a final extension at 72 °C for 10 min (Table 2). The PCR products were analyzed by 1% agarose gel electrophoresis and stained with ethidium bromide. The PCR products were sequenced using a BigDye 3.1 Terminator Sequencing Kit (Applied Biosystems) and an ABI Prism 3130 automated sequencer [26].

**Table 2:** List of primers used for PCR detection, sequencing and qRT-PCR assays

Gene target	Sequence (5′-3′)	Melting temp. (°C)	Amplicon length (bp)
16S rDNA	F: ACT AAC GCT GGC GGC GCG R: TAC CCA CGC TTT CGT GCC	56	1500
secY (1)	F: GCG ATT CAG TTT AAT CCT G R: GCG ATT CAG TTT AAT CCT GC	58	245
ORF22	F: ATT ATT GAG ACA GAG ATA R: AAG CGG AAC GGG ACG AAT	56	561
ORF36	F: AGT CGG GGT TCG ATA CTG R: GGT CAG TCC TGT AAC TGC	54	489
IS1500^a^	F: TAC GGA GCA AGA ACG GTT R: AGA TCC GAT TCT TAT GAT TC	56	143
IS1533^a^	F: TTG TAA TCC CTG TGT TGT TT R: TTA AAT AGT CCA CTC CTC G	56	130
secY (2)^a^	F: CTG AAT CGC TGT ATA AAA GT R: GAA GGC TGG TAA ACA AAA G	58	130

^a^for qRT-PCR

### Real-time PCR

Real-time PCR was performed in a total volume of 20 μl containing 50 ng of *Leptospira* spp. genomic DNA, 250 nM each of the forward and reverse primers, and 10 μl of 2× SYBR Green Master Mix (Applied Biosystems). The primer sequences and melting temperatures are described in Table 2. All of the reactions were performed in triplicate and were run on an ABI/PRISM 7500 Fast Sequence Detection System (Applied Biosystems) under the following conditions: 98 °C for 2 min, followed by 40 cycles of denaturation at 95 °C for 15 s and annealing/elongation for 15 s at 56 °C. DNA sequencing of the PCR products confirmed their specificity. The relative quantification of the IS1500 and IS1533 copy numbers was calculated by the 2^−ΔΔCt^ method [26] using the *secY* gene as the endogenous control [16]. The reference strain *L. interrogans* serovar Lai was used as the calibrator strain for the quantification of both IS1500 and IS1533. A BLAST search using each primer pair was performed to determine the number of amplifiable regions in the genome. In this analysis, we determined the presence of seven and two regions amplifiable by the primer pairs designed for IS1500 and IS1533, respectively.

### Molecular phylogenetic analysis

Homologous sequences of each genomic region of interest in other *Leptospira* spp. were retrieved from the GenBank database through a BLAST search [27]. This search was performed on the NCBI web server (http://blast.ncbi.nlm.nih.gov/Blast.cgi) by submitting the nucleotide sequences obtained from our isolates as queries against the refseq_genomic and refseq_rna databases. The search was conducted in October 2015. Then, we manually selected representative *Leptospira* species and serovar sequences (Additional files 2 and 3, according to the specific genomic region analyzed) from the BLAST results and conducted the phylogenetic analysis. The selected sequences together with the sequences obtained in this work were aligned using MUSCLE [28] implemented in MEGA 6 [29]. All of the phylogenetic trees in this work were inferred by the neighbor-joining method (model: maximum composite likelihood; bootstrap replications: 1000) implemented in MEGA 6 [29] and visualized using FigTree [http://tree.bio.ed.ac.uk/software/figtree/].

## Results

### Leptospirosis outbreaks from Minas Gerais were mainly caused by serovar Hardjo subtype Hardjoprajitno

The evaluation of the two leptospirosis outbreaks was performed using serological MAT to identify the prevalent *Leptospira* serogroups. A total of 132 out of 316 bovine serum samples from the first outbreak (41.8%) and 125 out of 258 samples from the second outbreak were MAT positive. Serovar Hardjo (Hardjoprajitno) showed a high positive reaction frequency [132 samples (41.8% of the positive samples) from the first outbreak and 125 samples (48.4% of the positive samples) from the second outbreak, followed by Wolffi (38.6–33.7%), Bratislava (35.1–33.7%) and Bataviae (21.8–24%). A small number of samples from the two leptospirosis outbreak reacted with serovars Canicola (two samples), Hebdomadis (five samples), Pyrogens (one sample) and Pomona (one sample). Analysis of the antibody titers showed that the highest titers at both farms were observed with serovar Hardjo (Additional file 1).

We isolated one *Leptospira* strain from the urine samples of seropositive animals from the first outbreak, which was referred to as “Norma”, and two strains from the second outbreak, which were referred to as “Lagoa” and “Bolivia”. The serological identification of these clinical isolates was assessed using two monoclonal antibodies (mAB) (F16C28 and F106C9, provided by the Royal Tropical Institute, Amsterdam, the Netherlands). For these analyses, MAT was applied to evaluate the agglutination titers of the monoclonal antibodies and clinical samples. The mAB F16C28 could distinguish *Leptospira* from serogroup Sejroe serovar Sejroe from *Leptospira* from serogroup Sejroe serovar Hardjo. The expected agglutination titer for serovar Sejroe was 80 and for serovar Hardjo was greater than 80 [30]. In contrast, mAB F106C9 showed a positive agglutination titer only for serovar Mini. The antibody analysis for all three isolates showed agglutination titers of 200 with mAB F16C28 and negative agglutination with mAB F106C9. These results indicated that Norma, Bolivia and Lagoa belonged to serogroup Sejroe serovar Hardjo.

### Phylogenetic analysis of the 16S rDNA and *secY* gene sequences

**Fig. 1:**
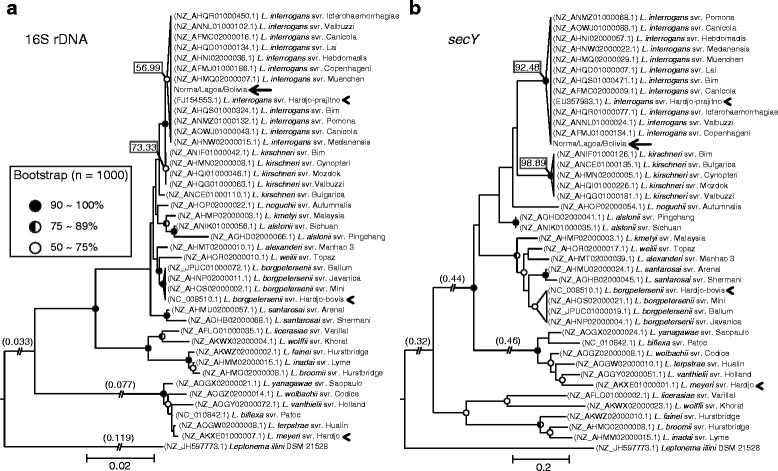
Phylogenetic trees of *Leptospira* spp. using (**a**) 16S rDNA and (**b**) *secY* sequences. The nucleotide sequences were aligned using Muscle, and the trees were generated using the neighbor-joining method (bootstrap: 1.000, model maximum composite likelihood). The clinical isolates from this work are indicated with *arrows*. *Leptospira* strains of the Hardjo serovar are indicated with *arrowheads*. *Circles* at nodes reflect the bootstrap support. An ancestral node without a circle indicates a bootstrap value below 50%. The bootstrap values of the nodes that group *L. interrogans* or *L. kirschneri* are located on these nodes in *rectangles*. Long branches are broken, and the real lengths are displayed on these branches in *parenthesis*. In both trees, orthologous sequences from *Leptonema illini* DSM 21528 were used as the outgroup

Because serovar Hardjo was encountered in three *Leptospira* species (*L. interrogans*, *L. borgpetersenii* and *L. meyeri*), we sequenced the 16S rDNA and *secY* gene genomic regions of our isolates (Norma, Lagoa and Bolivia) and applied phylogenetic methods for each genomic region to classify them taxonomically into one of these species. The sequences obtained from our isolates were identical to one another in both cases (16S rDNA and *secY*); thus, we sampled these sequences as one sample (Norma/Lagoa/Bolivia) in our phylogenetic analysis. In both phylogenetic trees (inferred by the neighbor-joining method; bootstrap: 10,000; model: maximum composite likelihood) generated using the 16S rDNA and *secY* sequences, all of the clinical isolates clustered together and were classified as *L. interrogans* (Fig. 1). In the 16S rDNA analysis, the cluster formed by *L. interrogans* serovars was supported by a low bootstrap value (56.99%) due to the high sequence similarity between the *L. interrogans* and *L. kirschneri* samples (90%–100%). In contrast, the phylogenetic analysis of the *secY* gene showed well-supported branches clustering both *L. interrogans* (bootstrap value: 92.48%) and *L. kirschneri* (bootstrap value: 98.89%) species, as shown in Fig. 1. Furthermore, the other *Leptospira* species of serovar Hardjo included in the analysis clustered together with their respective species in both phylogenetic trees. These results allowed us to identify our three isolates (Norma, Lagoa and Bolivia) as belonging to the *L. interrogans* species.

### Occurrence and molecular evolution of ORF22 and ORF36 of the *rfb* locus in *Leptospira* spp.

The *rfb* locus is largely used for the identification of different *Leptospira* serogroups. The ORF22 sequences of the *Leptospira* species of serovar Hardjo were highly similar to one another, whereas ORF36 was specific to *L. interrogans* serovar Hardjo [15]. To further classify our clinical isolates taxonomically, these ORFs were amplified by PCR. Reference strains of the *L. interrogans* and *L. borgpetersenii* species (Additional file 2) were used as the controls.

**Fig. 2:**
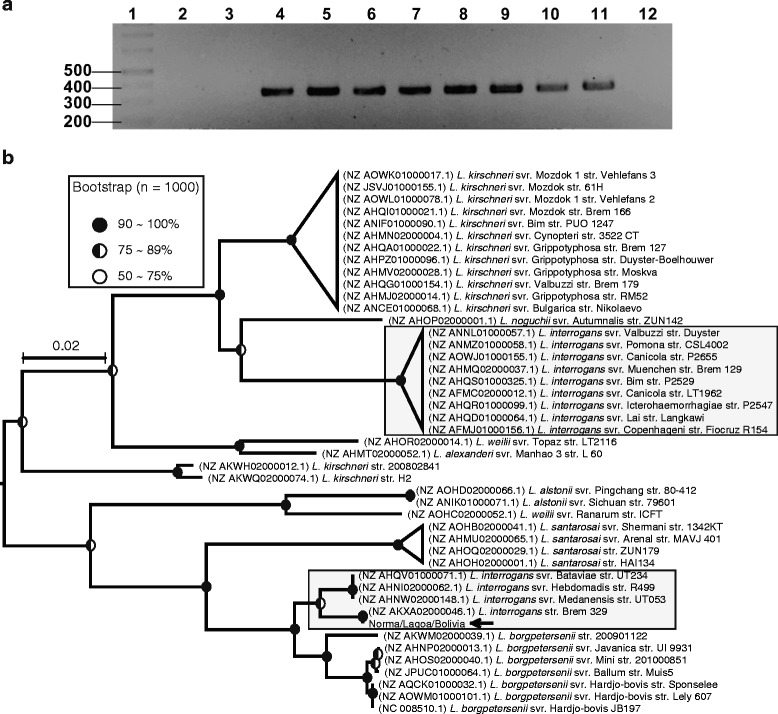
**a** PCR detection in *Leptospira* isolates and reference strains and (**b**) phylogenetic analysis of ORF22. The lanes in the gel (**a**) correspond to (1) molecular size marker, (2) svr. Lai, (3) svr. Copenhageni, (4) svr. Hardjo (OMS), (5) isolate Norma, (6) isolate Lagoa, (7) isolate Bolivia, (8) svr. Tarassovi, (9) svr. Mini, (10) svr. Hebdomadis, (11) svr. Sponselee and (12) negative control. The phylogenetic tree (**b**) was inferred using the neighbor-joining method (bootstrap: 1000, model: maximum composite likelihood) and rooted in the midpoint. The ORF22 sequences of the *L. interrogans* strains are separated into two clusters (*gray rectangle*). The *L. kirschneri* strains are separated into two clusters, and *L. borgpetersenii* is shown in one cluster. Samples representing our clinical isolates are indicated with *arrows*. *Circles* at nodes reflect the bootstrap support

For ORF22, PCR amplification with the primers Orf22–1 and Orf22–2 (Table 1) produced a primary 561-bp PCR product in samples with the genomic material of the *L. interrogans* serovars Hardjo, Hebdomadis, and Mini, the *L. borgpetersenii* serovar Tarassovi and the Hardjo strain Sponselee (Fig. 2a). Following the PCR amplification assay, the full-length ORF22 from all of the positive amplicons was sequenced. By retrieving orthologs of ORF22 from other *Leptospira* strains from the GenBank database, we observed that almost all of the *L. interrogans* strains in the database had ORF22 in their genomes. Nevertheless, the phylogenetic analysis demonstrated that some ORF22 sequences from *L. interrogans* strains clustered together with the ORF22 sequences from *L. borgpetersenii*, indicating that an ORF22 replacement event occurred in these *L. interrogans* strains (Fig. 2b). The clinical isolates (Norma/Lagoa/Bolivia), *L. interrogans* strain Brem 329 (NZ AKXA02000046.1) and serovars Bataviae (NZ AHQV01000071.1), Hebdomadis (NZ AHNI02000062.1) and Medanensis (NZ AHNW02000148.1) are examples of *L. interrogans* strains that contained ORF22 substitutions. Our data also showed two clades for the *L. kirschneri* bacterial strains, which suggested that this ORF had great potential for leptospiral molecular typing. The bootstrap values supporting the clade information from our clinical isolates and the retrieved sequences are shown in Fig. 2b.

**Fig. 3:**
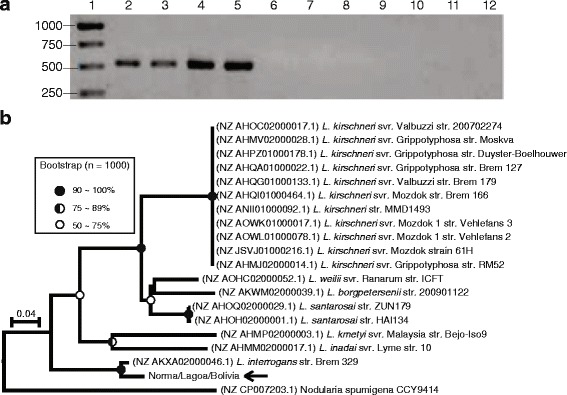
**a** PCR detection in *Leptospira* isolates and reference strains and (**b**) phylogenetic analysis of ORF36. The lanes in the gel (**a**) correspond to (1) molecular size marker, (2) svr. Hardjo (OMS), (3) isolate Norma, (4) isolate Lagoa, (5) isolate Bolivia, (6) svr. Lai, (7) svr. Copenhageni, (8) svr. Mini, (9) svr. Hebdomadis, (10) svr. Tarassovi, (11) svr. Sponselee and (12) negative control. The phylogenetic tree (**b**) was inferred using the neighbor-joining method (bootstrap: 1000, model: maximum composite likelihood) and rooted in the midpoint. Samples representing our clinical isolates are indicated with *arrows*. *Circles* at the nodes reflect the bootstrap support. An ancestral node without a *circle* indicates a bootstrap value below 50%

The occurrence of ORF36 in our isolates and the reference *Leptospira* strains was also characterized by PCR amplification and partial sequencing using the primer pair Orf36–1 and Orf36–2 (Table 2). In this case, positive amplification was observed only in the clinical isolates Norma, Bolivia and Lagoa and the reference strain OMS, which belonged to *L. interrogans* serogroup Sejroe serovar Hardjo (Fig. 3a). DNA sequencing and a BLAST search for similar sequences in the refseq genomic database also indicated the specificity of ORF36 to the Hardjoprajitno subtype. The BLAST search retrieved a single genome accession with high identity (96%) from *L. interrogans* strain Brem 329 (NZ_AKXA02000046.1). The other *Leptospira* genome accessions retrieved by the BLAST algorithm were from *L. borgpetersenii* (one sample), *L. kirschneri* (ten samples), *L. weilii* (1 sample), *L. santarosai* (two samples), *L. inadai* (one sample) and *L. kmetyi* (one sample), but their identities with the query were 74% (Fig. 3b). Despite the phylogenetic proximity of the *L. interrogans* and *L. kirschneri* species demonstrated in the 16S rDNA and *secY* gene sequences by the phylogenetic analysis (Fig. 1), the tree topology generated from ORF36 split the sequences from *L. interrogans* close to the root instead of clustering them together with the sequences from *L. kirschneri*. This finding could be an indication of gene acquisition by lateral gene transfer in these *L. interrogans* strains. However, we could also hypothesize that ORF36 underwent several mutation events and acquired a novel function while other *L. interrogans* strains lost this ORF.

The accession numbers of the sequences, the alignment positions and the predicted sequence information are shown in Additional file 2. All of the sequenced nucleotide sequences cited here were submitted to the GenBank database under accession numbers JQ765630.1-JQ765634 for 16S rRNA, KU2167449-KU216751/FJ667608 for *secY*, JQ965147- JQ965150 for ORF22 and JQ765636- JQ765639.1 for ORF36.

### Insertion sequence copy numbers in *Leptospira interrogans* serovars

Studies of IS1500 and IS1533 using the hybridization technique have suggested that the copy numbers of these mobile elements can be used as a tool for epidemiological purposes in strains classified as *L. interrogans* and *L. borgpetersenii*, respectively [31, 32]. In this study, we used quantitative real-time PCR to estimate and compare the copy numbers of the IS1500 and IS1533 insertion sequences in the *L. interrogans* strains classified as serovar Hardjo to enhance the molecular characterization of these clinical isolates. For copy number calibration, we used the *L. interrogans* serovar Lai strain 56,601 genome (NC_004342) and determined the number of amplifiable regions for each primer pair via sequence similarity analysis using the BLAST algorithm. These analyses determined that serovar Lai had seven and two amplifiable regions in its genome using the primer pairs for IS1500 and IS1533, respectively (Additional file 3).

**Fig. 4:**
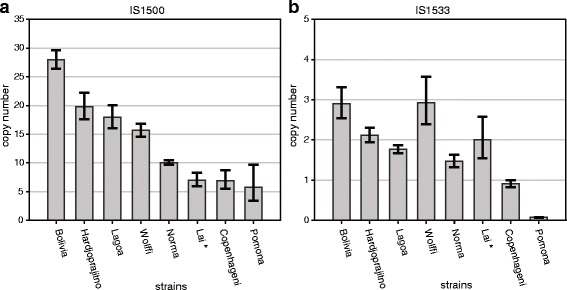
Copy number quantification of (**a**) IS1500 and (**b**) IS1533 by qRT-PCR in the *Leptospira* isolate and reference strain genomes. *Bars* represent the mean values of three replicates (±SD). IS copy number inference in other *Leptospira* spp. samples was performed using *secY* as the endogenous control and svr. Lai as the calibrator (*asterisk*)

For IS1500, *Leptospira* strains belonging to serogroup Sejroe (Norma, Lagoa, Bolivia, reference strain OMS and Wolffi) showed higher copy numbers (from ten to 28 copies) than the other two serogroups [Icterohaemorrhagiae (serovars Lai and Copenhageni) and Pomona]. In serogroup Sejroe, strains belonging to serovar Hardjo (Norma, Lagoa, Bolivia and OMS) showed large variations in their copy numbers (Fig. 4). Our results determined the existence of at least ten copies for strain Norma, 28 for strain Bolivia, 18 for strain Lagoa and 20 for reference strain OMS. The sample from serovar Wolffi showed a copy number comparable to the Lagoa strain, with 16 copies. In the other *L. interrogans* isolates, we identified 7 copies in the Copenhageni strain and 6 copies in the Pomona strain (Fig. 4).

In contrast to IS1500, the IS1533 mobile element analysis demonstrated little variation in the copy numbers. In serovar Hardjo, at least three copies were present in Bolivia and two copies in Lagoa, Norma and the reference strain OMS. Of the other *L. interrogans* strains, at least three copies were present in serovar Wolffi, one copy in serovar Copenhageni and no copies in serovar Pomona. The standard deviation represented the Ct value analysis from three independent experiments, as show in Fig. 4.

To check the consistency of our results, we also retrieved the genome sequence of *L. interrogans* serovar Copenhageni from the GenBank database (NC_005823.1) and verified whether the copy number obtained by each primer pair determined by real-time PCR in this serovar matched the number of amplifiable regions determined in silico by the BLAST search. We identified seven amplifiable regions using the IS1500 primer pair as the queries and a single amplifiable region using the IS1533 primer pair as the queries (Additional file 3). These results corroborated the results obtained from the real-time PCR analysis.

## Discussion

MAT is considered the standard serological test for leptospirosis diagnosis, but cross-reactivity frequently occurs, which raises questions concerning correct serovar identification [22]. Although the isolation of *Leptospira* spp. from infected animals or patients and molecular characterization are accurate diagnostic procedures for the taxonomic and serovar identification of a *Leptospira* strain, these procedures are laborious and expensive.

Another issue with leptospirosis serovars is that their classification based on genomic regions often does not correspond to their classification based on serological analysis [2]. This lack of correspondence prevents serological correlations because the genomic regions selected for the analysis represent ribosomal regions and housekeeping genes. To overcome this limitation, approaches based on molecular biology techniques, including restriction fragment length polymorphism (RFLP) analysis [33] and multiple locus sequence typing (MLST;[34]), have been proposed to render the serovar identification procedure more rapid and less expensive. Additionally, PCR-based approaches using variable number tandem repeats (VNTR; [35]) and the *rfb* locus [36] have demonstrated promise in distinguishing *Leptospira* serovars. Here, we characterized two leptospirosis outbreaks at dairy farms in the state of Minas Gerais, Brazil, using a conventional serological analysis (MAT) in combination with molecular biology approaches.

Serological analysis using MAT in a total of 642 animals from two dairy farms in the state of Minas Gerais determined that approximately half of the animals were seropositive and that Hardjo was the prevalent serovar. We also observed cross-reactivity between the Hardjo and Wolffi serovars, which could be explained by both serovars belonging to the Sejroe serogroup [37]. Previous bovine leptospirosis epidemiological studies of livestock from different Brazilian regions have also shown the seroprevalence of the Hardjo serovar [38, 39], demonstrating a high incidence of this serovar in leptospirosis outbreaks in Brazil.

The three clinical isolates obtained in this work (Norma, Lagoa and Bolivia) were classified as the Sejroe serogroup by the monoclonal antibody analysis and as *L. interrogans* species by the phylogenetic analysis of the 16S rDNA and *secY* gene sequences. We also achieved a more detailed taxonomic classification by analyzing ORF22 and ORF36 of the *rfb* locus. All of the clinical isolates contained an ORF22 with high similarity to the reference strain Hardjoprajitno and positive amplification of ORF36, which is a specific ORF encountered in *L. interrogans* serovar Hardjo. Due to the high specificity of these molecular markers for subtype Hardjoprajitno, we classified all of the clinical isolates as belonging to *Leptospira interrogans* serogroup Sejroe serovar Hardjo based on the serological and molecular data.

The *rfb* locus was previously described in different works as a genomic region acquired by *Leptospira* spp. from lateral gene transfer. Thus, we presume that differences can occur among bacterial strains, especially in microorganisms isolated from different geographical areas and from different hosts.

Previous studies have described sequence dissimilarities in some ORFs of the *rfb* locus between *L. interrogans* serovar Copenhageni and *L. interrogans* serovar Hardjo, including ORF22 and ORF36 [14, 15]. In the PCR analysis, amplification using our primers for ORF22 was observed in serovars classified in the serogroups Sejroe, Hebdomadis, Mini and Tarassovi. Conversely, the Lai, Copenhageni and Canicola serovars were negative in this analysis. Using sequencing and phylogenetic methods, we observed that the PCR-positive serovars were clustered in a branch with the *L. interrogans* and *L. borgpetersenii* species. Although the Lai, Copenhageni and Canicola serovars were PCR-negative for our primers, ORF22 was present in their genomes and clustered in another branch with only the *L. interrogans* species (Fig. 2b). The tree topology clearly demonstrated a lateral gene transfer event in which the ORF from *L. borgpetersenii* replaced the ORF22 of some *L. interrogans* species. The phylogenetic tree also showed a close relationship between the ORF22 sequences from our isolates from serogroup Sejroe serovar Medanensi and the Hebdomadis and Mini serogroups. Historically, the Hebdomadis, Mini and Sejroe serogroups belonged to the same serogroup (Hebdomadis) and displayed a similar antigenic profile [40]. Our data from the ORF22 analysis were in disagreement with previous studies related to the *rfb* locus in *L. interrogans* serovar Hardjo because our isolates were similar to *L. interrogans* serovar Hebdomadis. This finding suggested that ORF22 might play an important role in shaping the antigenic profiles of these serogroups because they had the same origin.

ORF36 was previously described as an extra ORF present in *L. interrogans* serovar Hardjo [15]. PCR analysis of this ORF demonstrated positive amplification only in the *L. interrogans* serovar Hardjo reference strain and our clinical isolates (Norma, Bolivia and Lagoa), as show in Fig. 3a. To evaluate the distribution of the ORF36 sequence throughout the available *Leptospira* genomes, we queried this ORF against the NCBI genome database. This query retrieved a single strain of the *L. interrogans* species with high similarity (Brem 329) and other *Leptospira* species with low similarity, including *L. borgpetersenii*, *L. kirschneri*, *L. weilii*, *L. santarosai*, *L. inadai* and *L. kmetyi* (Fig. 3b). In the phylogenetic analysis, the tree topology generated by the ORF36 sequences was contrasted with the topologies generated by the 16S rDNA and *secY* sequences when we compared the closeness between the *L. interrogans* and *L. kirschneri* samples, which again suggested an evolutionary event that escaped the neutral models. ORF36 was likely deleted early in the *L. interrogans* lineage and was acquired later by horizontal gene transfer in some species. We could also hypothesize that this ORF was subfunctionalized and deleted in almost all of the *L. interrogans* serovars, whereas the other serovars neofunctionalized ORF36 and maintained it in their genomes. Here, our analyses are in agreement with previous discussions of the *rfb* locus and indicate that this ORF may be unique to *L. interrogans* serovar Hardjo strains. We also emphasize the high similarity between the ORF22 and ORF36 sequences of our clinical isolates and *L. interrogans* strain Brem 329. The latter strain was isolated in Germany from horses and was classified in the Sejroe serogroup but had no serovar classification. Based on our analysis, we suggest that strain Brem 329 also belongs to the Hardjo serovar, but this suggestion requires serological analysis for confirmation. Here, we designed primers to search for the genomic regions described in a previous work [14] that discussed *L. interrogans* serovar Hardjo and showed that this bacterial type had the *rfb* locus obtained from *L. interrogans* serovar Copenhageni and *L. borgpetersenii* serovar Hardjobovis. In this work, we searched for this information in our isolates because a comparison of bacterial strains obtained from different geographic regions worldwide could reveal different genomics features. We designed primers for these regions, sequenced these specific regions and compared the sequences with the available data. Our analysis showed that ORF22 in our isolates was similar to ORF22 in *L. interrogans* serovar Hebdomadis, which differed from the data described by De La Peña et al., and that ORF36 could be found in other *L. interrogans* serovars. Previous works related to the *rfb* locus theme mentioned that this genomic region was acquired from bacteria classified in this genus. Based on these statements, we attempted to verify the presence of these genes in our isolates.

Next, we performed an IS analysis of the *Leptospira* reference strains and our samples. In this bacterial phylum, mobile elements represent an important region that is directly involved in genomic diversity among microorganisms [41, 42]. Moreover, information about IS copy numbers in the genome has been demonstrated to be useful in epidemiological studies of the *Leptospira* genus [26, 31, 43]. We found considerable variation in the copy numbers among the serovars and even among isolates of the same serovar, which allowed us to identify each strain by the IS copy number profile [21, 31, 32, 44]. One methodology used to determine the IS copy number is Southern blotting using IS fragments as probes [21, 31, 32]. Here, we recommend quantitative real-time PCR as an alternative to determine the copy number of a mobile element with greater precision. We selected the insertion sequences IS1500 and IS1533, which are used for the taxonomic and serovar classification of *L. interrogans* and *L. borgpetersenii*, respectively [31, 32]. The copy number estimations determined here were shown to be consistent, with the number determined in the Copenhageni strain almost the same as the number determined by the in silico analysis using the genome sequence of this strain.

The quantitative PCR assays for IS1500 demonstrated significant copy number variation among the *L. interrogans* serovars (copy number range: 6 to 28) and even among our clinical isolates (copy number range: 10 to 28). From these results, we classified the samples containing more IS1500 copies as belonging to the Sejroe serogroup and the samples with seven or six copies as belonging to the Icterohaemorrhagiae or Pomona serogroups. Experiments with more samples are needed to determine how well the quantification of IS1500 can distinguish among *L. interrogans* serovars or serogroups, but the large variation in the IS1500 copy number and good precision of the method in determining the copy number are indicative of its potential for epidemiological studies. IS1533 is well distributed in the *L. borgpetersenii* genome and can reach almost one hundred copies; in contrast, this mobile element is present in *L. interrogans* at a low copy number, as indicated in previous studies. Our results showed a low number and variation of IS1533 among the sampled *L. interrogans* serovars (copy number range: 0 to 4), suggesting that the transference and recombination of genetic material between the two species occurred in punctual regions of the *L. interrogans* genome and might not have interfered with its IS1533 copy number. Moreover, the difference in the IS contents of the Lagoa and Bolivia strains isolated at the same time represent clear genetic variability that suggests the co-circulation of different strains during an outbreak.

*Leptospira* spp. identification is an essential procedure to aid in the selection of the best means to control the disease. In this work, we endorse the importance of molecular characterization for the identification of *Leptospira* spp. by analyzing ORF22 and ORF36 from the *rfb* locus and the copy numbers of mobile elements (IS1500 and IS1533). Phylogenetic analysis based on *rfb* locus genes suggested the great potential of this region for the generation of taxonomic information. IS analysis also helps address other questions concerning the genomic organization of *Leptospira* strains because differences in copy numbers may be associated with genetic rearrangements and pseudo-gene formation. Finally, we succeeded in isolating three *Leptospira* samples from livestock from the state of Minas Gerais, Brazil, which were classified as *L. interrogans* serogroup Sejroe serovar Hardjo. Therefore, this study enhanced epidemiological research and suggested different vaccine formulations to contribute to bovine leptospirosis control in this endemic region. Research to analyze distinct genomic features among *Leptospira* spp. using molecular approaches and bioinformatics studies should be undertaken to improve our knowledge of their pathogenicity, evolution and epidemiology.

## Conclusion

The present study described the occurrence of *L. interrogans* serovar Hardjo subtype Hardjoprajitno in livestock production through the isolation of bacterial strains. Partial genetic analysis using *rfb* locus genes (ORF22 and ORF36) suggested that these genes could be useful for taxonomic studies. Genetic comparative analyses among *Leptospira* spp. propose differences among clinical strains, which may contribute to new studies within these microorganisms. Moreover, this work may improve knowledge about the most prevalent serovar in Brazilian herds of cattle. An efficient vaccine should contain local isolates, and periodic molecular analysis of circulating isolates may contribute to the identification of important genetic variants that should be included in new vaccine formulations for leptospirosis control.

## Additional files

Additional file 1: *Leptospira* spp. strains used in MAT and PCR analysis. (XLS 34 kb)

Additional file 2: Sequences included in molecular phylogenetic analysis (GenBank access). (XLS 43 kb)

Additional file 3: Blast analysis for qRT-PCR assay for IS1500, IS1533 and *secy*. (XLS 100 kb)
